# Prevalence of Musculoskeletal Disorders Among People Living With Type 2 Diabetes in the Western Region of Saudi Arabia: A Cross-Sectional Study

**DOI:** 10.7759/cureus.91111

**Published:** 2025-08-27

**Authors:** Ashwaq S Althobaiti, Shomoq J Alkhadidi, Shumoukh A Alhumyani, Abdulmajeed Algethami

**Affiliations:** 1 Internal Medicine, Taif University, Taif, SAU

**Keywords:** diabetes, disorders, msk, prevalence, saudi, western

## Abstract

Introduction: Diabetes is a multi-systemic disease allowing for various symptoms, including musculoskeletal (MSK) disorders, and the aim was to assess the prevalence of MSK disorders among 539 type 2 diabetic patients in the Western region of Saudi Arabia.

Methods: A descriptive cross-sectional study was conducted in the Western region of Saudi Arabia. Data were collected through an online questionnaire that assessed patients’ demographics, medical histories, and frequency of general and particular MSK problems associated with diabetes mellitus.

Results: The median age of the patients was 30 years, with 335 (62.2%) females. The median BMI was 24.8 kg/m², and 118 (21.9%) were obese. Most patients had diabetes for 1-10 years (366, 67.9%), and 208 (38.6%) had uncontrolled HbA1c levels (>7%). Only 162 (30.1%) reported receiving education regarding the relationship between diabetes and MSK health. Macrovascular complications were observed in 50 patients (9.3%), most commonly peripheral arterial disease (26, 52%), while 129 (23.9%) had other diabetes-related complications. Overall, 172 patients (31.9%) had at least one MSK disorder, including trigger finger (22, 4.1%), osteoarthritis (90, 16.7%), carpal tunnel syndrome (50, 9.3%), diabetic cheiroarthropathy (50, 9.3%), shoulder capsulitis (50, 9.3%), limited joint mobility (43; 8%), Charcot joint (16; 3%), Dupuytren’s contracture (36; 6.7%), diabetic myopathy (31; 5.8%), and diabetic osteoporosis or other diabetes-related bone disorders (75, 13.9%). Multivariate logistic regression identified the presence of diabetes-related complications and diabetic foot as significant risk factors for MSK disorders.

Conclusion: Early diagnosis of MSK disorders in diabetic patients is crucial for timely and effective therapy.

## Introduction

The chronic metabolic condition known as diabetes mellitus, or DM for short, is characterized by high blood sugar levels that are caused by the body's inability to generate or use insulin [1]. In 2000, the prevalence of diabetes was estimated to be 2.8% worldwide; by 2030, it is expected to increase to 4.4% across all age groups. The number of people with diabetes would increase from 171 million in 2000 to 366 million by 2030 [1].

Diabetes is the primary cause of several health issues, including retinopathy, peripheral neuropathy, renal failure, and vascular disease. Other symptoms, such as musculoskeletal (MSK) issues, may appear because of its multi-systemic nature [1,2]. DM has been associated with a number of MSK disorders that can cause significant disability. These conditions include Dupuytren's contracture (DC), shoulder capsulitis, osteoarthritis (OA), trigger finger (TF), carpal tunnel syndrome (CTS), decreased joint mobility, and other abnormal outcomes [2].

Exactly 46.7% of the 270 participants in a 2019 study with type 2 diabetes exhibited MSK issues, such as hand stiffness syndrome (10%), flexor tenosynovitis (10.4%), frozen shoulder (20.7%), and decreased joint mobility (9.6%). There were significant relationships between the length of diabetes (p = 0.003, 0.0001), glycemic control (p = 0.004, 0.001), and hand stiffness syndrome, frozen shoulder, and limited joint mobility. Medication was used to treat just 42.9% of the issues that were diagnosed. The study recommends medication as a way to improve quality of life and emphasizes the importance of periarticular evaluation in diabetic individuals with uncontrolled diabetes [3].

A study conducted by Kalam et al. [4] found that MSK abnormalities were more common in women than men and occurred frequently among individuals with type 2 diabetes. Lower limb pain was the most prevalent, and some moderate chair mobility problems were evident. Fewer respondents said they had problems opening prescription bottles and walking. Most patients were able to carry on with their normal lifestyles without any problems. Higher hyperglycemia levels were linked to increased challenges in doing daily tasks. The study suggests controlling blood sugar levels and promoting physical therapy through diabetes awareness campaigns as ways to prevent and treat these diseases. This indicates a variation in the results of the previous research, as well as a few sample size statistics [4].

Although the prevalence of MSK complaints is important, it has not been thoroughly studied in patients with type 2 diabetes in the Western region of Saudi Arabia. Therefore, the primary aim of our study was to determine the prevalence of MSK complaints among type 2 diabetic patients in this region, while secondary aims included identifying factors associated with these complaints.

## Materials and methods

Study design, location, and time frame

A descriptive cross-sectional study was conducted in the Western region of Saudi Arabia, including these cities (Taif, Makkah, Jeddah, Al Madinah, and Yanbu) in the time from June to September 2024.

Study participants

The inclusion criteria were type 2 diabetic patients who have been diagnosed with the disease for at least one year, aged ≥18 years, in the Western Region of Saudi Arabia. The exclusion criteria were patients having a history of hand trauma, arthroplasty, chronic rheumatic diseases, end-stage renal disease, thyroid disorders, and people who were unwilling to answer the questionnaire from the study.

Sampling technique

The sample size was calculated at 377 persons by Raosoft, Inc. (Seattle, WA), using the following formula and applying means and standard deviation, taking into account the standard deviation (1.96) for the 95% confidence interval and the maximum allowable marginal error (0.05). As a result, the computed minimum sample size for this study is n = (1.96) 2x 0.50x 0.50 / (0.50) 2 = 384 participants.

Data collection

Based on a thorough analysis of prior research on the assessment of the prevalence of musculoskeletal diseases among individuals with DM, an online questionnaire was created [2,5]. The purpose of the questionnaire was to get pertinent data and perspectives from the respondents. A thorough review process was used on the questionnaire to guarantee its validity and reliability. Two impartial experts who were neither authors nor participants in the research reviewed the questionnaire externally. These professionals are well-versed in the field of endocrinology and have a wealth of expertise. The study was conducted via the Google online survey platform, and the chosen individuals will receive the questionnaire at random via social media apps. Participants will be required to provide informed permission and get a thorough explanation of the study's purpose prior to beginning the survey. The first section of the questionnaire asks about the participants' age, gender, educational background, marital status, and employment situation. The participants' medical histories are the main topic of the survey's second section. It also asks about prior schooling. The frequency of both general and particular MSK problems associated with DM was examined in the third and fourth sections.

Before the primary data gathering stage, a pilot study was carried out to confirm the viability and efficacy of our research approach. As a test run, the pilot study made it possible to spot any possible problems or areas in which the questionnaire, data collection methods, and study design needed to be improved. A small sample of people who met the study's inclusion criteria was included in the pilot study. A basic random sample consisting of roughly 20 individuals was gathered. After completing the questionnaire, these participants were asked to comment on its comprehensiveness, relevance, and clarity. After the pilot study was over, the participant comments were examined, and the questionnaire underwent any required changes to guarantee its validity and reliability. The questionnaire's quality was increased through this iterative procedure, which also improved the study's overall design. Cronbach's alpha test was used to assess the internal consistency of the questionnaire items, and the estimated result was more than 0.7, ensuring the survey's internal validity. The survey instrument's coherence and reliability were assessed statistically using the Statistical Product and Service Solutions (SPSS, version 26; IBM SPSS Statistics for Windows, Armonk, NY) program. Items with low item-to-total correlations were flagged and may be removed. A group of experts and healthcare providers with knowledge in the relevant subject was also given the survey.

Ethical considerations

An ethical approval for the study was obtained from Taif University Scientific Research Ethics Committee, Taif University, Saudi Arabia, with no. (HAO-02-T-105). Accordingly, ethical approval was granted (from December 23) for one year only.

Data analysis

Data were statistically analyzed using the SPSS application. To investigate the association between the variables, the chi-squared test (χ2) was applied to qualitative data that were expressed as numbers and percentages. The association between the quantitative non-parametric variables that were expressed as median and interquartile range (IQR) was examined using the Mann-Whitney test. Multivariate logistic regression analysis was done to assess factors associated with MSK disorders among the studied patients, where the odds ratio (OR) was calculated at a 95% confidence interval (CI). A p-value of less than 0.05. was considered statistically significant.

## Results

Table 1 and the Appendix show that the median age of the 539 diabetic patients studied was 30 years. Among them, 335 (62.2%) were female, 531 (57.7%) held a bachelor’s degree, 180 (33.4%) were employed, and 263 (48.4%) were single. The median BMI was 24.8 kg/m², with 118 (21.9%) classified as obese.

**Table 1:** Distribution of studied diabetic patients according to their demographic characters and BMI IQR = Interquartile range, BMI = Body Mass Index

Variable	No. (%)
Age (years) (Median - IQR)	(30 - 27)
Gender	
Female	335 (62.2)
Male	204 (37.8)
Educational Level	
Illiterate	29 (5.4)
High school or below	138 (25.6)
Diploma or equivalent	30 (5.6)
Bachelor's degree	311 (57.7)
Master's degree	22 (4.1)
PhD degree	9 (1.7)
Occupation	
Student	184 (34.1)
Unemployed	116 (21.5)
Retired	59 (10.9)
Employed	180 (33.4)
Marital Status	
Widowed	18 (3.3)
Single	263 (48.8)
Married	239 (44.3)
Divorced	19 (3.5)
BMI (kg/m^2^) (Median - IQR)	(24.8 - 7.64)
BMI Categories	
Underweight	48 (8.9)
Normal weight	230 (42.7)
Overweight	143 (26.5)
Obese	118 (21.9)

Table 2 shows that most patients had a duration of DM of 1-10 years (366, 67.9%), with 208 (38.6%) having an uncontrolled glycated hemoglobin (HbA1c) level (>7). Only 162 (30.1%) reported receiving education or information about the relationship between diabetes and musculoskeletal health. Among the patients, 50 (9.3%) had macrovascular complications, with peripheral arterial disease (PAD) (26, 52%) and coronary artery disease (CAD) (20, 40%) being the most common. Regarding DM-related complications, 129 (23.9%) reported having any complications, with dyslipidemia (66, 51.1%), retinopathy (48, 37.2%), and diabetic foot (33, 25.5%) being the most prevalent.

**Table 2:** Medical history of the studied diabetic patients PAD = Peripheral Arterial Disease, CAD = Coronary Artery Disease, DM = Diabetes Mellitus

Variable	No. (%)
Duration of DM (Years)	
<1	84 (15.6)
1-10	366 (67.9)
11-20	61 (11.3)
21-30	18 (3.3)
>30	10 (1.9)
HbA1c Level	
Uncontrolled (>7)	208 (38.6)
Controlled (<7)	215 (39.9)
Don’t know	116 (21.5)
Have you received any education or information about the relationship between diabetes and musculoskeletal health?	
No	217 (40.3)
Yes	162 (30.1)
Presence of macrovascular complications	
No	489 (90.7)
Yes	50 (9.3)
If yes, specify: (No.: 50)	
Coronary Artery Disease (CAD)	20 (40)
Peripheral Arterial Disease (PAD)	26 (52)
Stroke	10 (20)
Aortic aneurysm	8 (16)
DM Complications	
No	410 (76.1)
Yes	129 (23.9)
If yes, specify: (No.: 129)	
Dyslipidemia	66 (51.1)
Nephropathy	12 (9.3)
Neuropathy	24 (18.6)
Retinopathy	48 (37.2)
Diabetic foot	33 (25.5)

**Figure 1:**
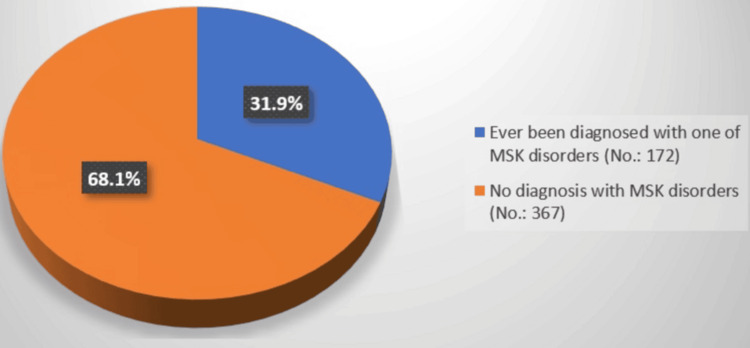
Percentage distribution of ever been diagnosed with any of the MSK disorders MSK = Musculoskeletal

Table 3 illustrates the prevalence of MSK complications among the studied diabetic patients. OS had the highest prevalence (90, 16.7%), while Charcot joint had the lowest (16, 3%). Carpal tunnel syndrome and cheiroarthropathy each had a prevalence of 9.3% (50), Dupuytren’s contracture of 6.7% (36), and trigger finger of 4.1% (22). Additionally, limited joint mobility was observed in 8% (43) of patients, shoulder capsulitis in 9.3% (50), diabetic myopathy in 5.8% (31), and diabetic osteoporosis or other diabetes-related bone issues in 13.9% (75). Figure 1 shows that the overall prevalence of any MSK disorder was 31.9% (172).

**Table 3:** Distribution of MSK complications among the studied diabetic patients

Variable	No. (%)
Have you ever been diagnosed with osteoarthritis?	
No	449 (83.3)
Yes	90 (16.7)
Have you ever been diagnosed with Carpal Tunnel Syndrome? A disorder that affects the hand and wrist, resulting from compression of the median nerve, and can cause symptoms such as tingling, numbness, and weakness in the affected hand.	
No	487 (90.4)
Yes	50 (9.3)
Have you ever been diagnosed with Dupuytren's contracture? This condition causes one or more fingers to bend into the palm, making it difficult to straighten them.	
No	503 (93.3)
Yes	36 (6.7)
Have you ever been diagnosed with Cheiroarthropathy? thickening and tightening of the skin on the hands, leading to reduced mobility and flexibility.	
No	489 (90.7)
Yes	50 (9.3)
Have you ever been diagnosed with trigger finger? Trigger finger, also known as stenosing tenosynovitis, is a condition where one of your fingers gets stuck in a bent position and may straighten with a snap, like pulling and releasing the trigger of a gun.	
No	517 (95.9)
Yes	22 (4.1)
Have you ever been diagnosed with limited joint mobility? Limited joint mobility is a condition characterized by restricted movement in one or more joints, commonly observed in individuals with diabetes mellitus. Affects the hands and fingers, accompanied by stiffness, and reduced flexibility.	
No	496 (92)
Yes	43 (8)
Have you ever been diagnosed with Charcot joint? This is a deformity and functional impairment, often occurring in the foot or ankle due to nerve damage and loss of sensation in a joint.	
No	523 (97)
Yes	16 (3)
Have you ever been diagnosed with shoulder capsulitis?	
No	489 (90.7)
Yes	50 (9.3)
Have you ever been diagnosed with diabetic myopathy? There is weakness, atrophy, and changes in muscle structure and function.	
No	508 (94.2)
Yes	31 (5.8)
Have you ever been diagnosed with diabetic osteoporosis or any diabetic-related bone issues?	
No	464 (86.1)
Yes	75 (13.9)

Table 4 shows that the prevalence of MSK disorders was significantly higher among patients with an older mean age (38.09 ± 17.08 years), those who were married, and those with a higher mean BMI, including overweight patients (p < 0.05).

**Table 4:** Relationship between the prevalence of MSK disorders and participants’ demographics and BMI IQR = Interquartile Range, BMI = Body Mass Index, MSK = Musculoskeletal p-value is considered significant at <0.05.

Variable	MSK disorders		χ2	p-value
	Present No. (%)	Absent No. (%)		
Age (years) (Median - IQR)	(34.5 - 28)	(29 - 28)	2.02	0.042
Gender				
Female	97 (56.4)	238 (64.9)	3.55	0.059
Male	75 (43.6)	129 (35.1)		
Educational Level				
Illiterate	11 (6.4)	18 (4.9)	1.88	0.685
High school or below	47 (27.3)	91 (24.8)		
Diploma or equivalent	11 (6.4)	19 (5.2)		
Bachelor's degree	94 (54.7)	217 (59.1)		
Master's degree	7 (4.1)	15 (4.1)		
PhD degree	2 (1.2)	7 (1.9)		
Occupation				
Student	46 (26.7)	138 (37.6)	7.41	0.06
Unemployed	45 (26.2)	71 (19.3)		
Retired	18 (10.5)	41 (11.2)		
Employed	63 (36.6)	117 (31.9)		
Marital Status				
Widowed	8 (4.7)	10 (2.7)	9.1	0.028
Single	69 (40.1)	194 (52.9)		
Married	90 (52.3)	149 (40.6)		
Divorced	5 (2.9)	14 (3.8)		
BMI (kg/m^2^) (Median - IQR)	(25.71 - 6.38)	(24.45 - 8)	2.23	0.025
BMI Categories				
Underweight	6 (3.5)	42 (11.4)	13.37	0.004
Normal weight	71 (41.3)	159 (43.3)		
Overweight	58 (33.7)	85 (23.2)		
Obese	37 (21.5)	81 (22.1)		

Table 5 shows that the prevalence of MSK disorders was significantly higher among patients with a DM duration of 1-10 years and those with any macrovascular complications, except stroke (p < 0.05). Additionally, MSK disorders were significantly more prevalent among patients with any DM-related complications (p < 0.05).

**Table 5:** Relationship between the prevalence of MSK disorders and participants’ medical history PAD = Peripheral Arterial Disease, CAD = Coronary Artery Disease, DM = Diabetes Milletus, MSK = Musculoskeletal p-value is considered significant at <0.05.

Variable	MSK disorders		χ2	p-value
	Present No. (%)	Absent No. (%)		
Duration of DM (years)				
<1	12 (7)	72 (19.6)	17.3	0.002
1-10	130 (75.6)	236 (64.3)		
11-20	22 (12.8)	39 (10.6)		
21-30	7 (4.1)	11 (3)		
>30	1 (0.6)	9 (2.5)		
HbA1c level				
Uncontrolled (>7)	64 (37.2)	144 (39.2)	1.63	0.441
Controlled (<7)	75 (43.6)	140 (38.1)		
Don’t know	33 (19.2)	83 (22.6)		
Have you received any education or information about the relationship between diabetes and musculoskeletal health?				
No	72 (41.9)	145 (39.5)	0.26	0.604
Yes	100 (58.1)	222 (60.5)		
Presence of macrovascular complications				
No	135 (78.5)	354 (96.5)	44.93	<0.001
Yes	37 (21.5)	13 (3.5)		
If yes, specify: (No.: 50)				
Coronary artery disease (CAD)	15 (8.7)	5 (1.4)	17.74	<0.001
Peripheral arterial disease (PAD)	21 (12.2)	5 (1.4)	30.01	<0.001
Stroke	6 (3.5)	4 (1.1)	3.7	0.054
Aortic aneurysm	7 (4.1)	1 (0.3)	11.54	0.001
DM Complications				
No	78 (45.3)	332 (90.5)	13.93	<0.001
Yes	94 (54.7)	35 (9.5)		
If yes, specify: (No.: 129)				
Dyslipidemia	49 (28.5)	17 (4.6)	12.02	<0.001
Nephropathy	10 (5.8)	2 (0.5)	14.93	<0.001
Neuropathy	18 (10.5)	6 (1.6)	21.46	<0.001
Retinopathy	34 (19.8)	14 (3.8)	16.73	<0.001
Diabetic foot	29 (16.9)	4 (1.1)	15.67	<0.001

Table 6 shows that multivariate logistic regression analysis was conducted to assess the risk factors (independent predictors) of MSK disorders among the studied patients. The analysis found that having any DM complications or having diabetic foot were significant risk factors (independent predictors) of MSK disorders among the patients.

**Table 6:** Multivariate logistic regression analysis of risk factors of MSK disorders among studied patients MSK = Musculoskeletal p-value is considered significant at <0.05.

Variable	B	Wald	p-value	Odds Ratio (CI:95%)
Age (years) (Median - IQR)	0.01	0.02	0.879	0.9 (0.98-1.01)
Marital Status	0.32	2.28	0.13	0.72 (0.47-1.1)
BMI (kg/m^2^) (Mean ± SD)	0.02	0.43	0.51	1.02 (0.94-1.12)
BMI categories	0.13	0.21	0.647	0.87 (0.5-1.53)
Duration of DM (years)	0.02	0.03	0.861	1.02 (0.75-1.39)
Presence of macrovascular complications	0.6	0.91	0.339	0.54 (0.16-1.87)
Coronary artery disease (CAD)	0.58	0.53	0.443	0.79 (0.4-1.24)
Peripheral arterial disease (PAD)	0.6	0.62	0.426	0.83 (0.4-1.27)
Aortic aneurysm	0.48	0.14	0.7	0.62 (0.13-1.45)
Presence of DM complications	1.37	8.64	0.003	1.25 (1.1-3.67)
Dyslipidemia	0.8	3.05	0.08	0.23 (0.9-1.99)
Nephropathy	1.45	2.55	0.11	0.27 (0.72-1.98)
Neuropathy	0.27	0.19	0.655	0.76 (0.23-2.52)
Retinopathy	0.31	0.44	0.507	0.36 (0.54-1.43)
Diabetic foot	1.63	6.39	0.011	5.14 (1.44-8.31)

## Discussion

In this study, we investigated the prevalence of MSK symptoms in individuals with diabetes in the Western Region of Saudi Arabia. According to the current study, MSK issues affect 31.9% of diabetic individuals. In a prior comprehensive analysis, the prevalence of all forms of MSDs among individuals with diabetes was 58.15%, indicating a decreased prevalence of MSK problems [5].

OA is the most common MSK symptom, occurring in 16.7% of cases. Numerous studies have shown that OA is a typical sign of diabetes [6-8]. In one of these studies, AlOayan et al. found that 71.3% of DM patients had OA [7]. Another study conducted in Ethiopia by Abebe et al. reported that 23.29% of participants had MSK disorders [9].

The second most often mentioned ailment was carpal tunnel syndrome. CTS was identified as a predicted adverse effect of DM in a number of investigations [10]. According to a recent survey, 8.4% of people had CTS. This study found a frequency of 9.3% [7]. The prevalence of CTS was found to be around 3% in the general population and 14% in diabetic patients, with the prevalence rising to 30% [11] in the presence of diabetic polyneuropathy (DPN).

Reduced hand joint mobility, fixed flexion contracture at the metacarpophalangeal (MCP), proximal interphalangeal (PIP), and distal interphalangeal (DIP) joints, as well as palmar and dorsal surface sclerotic changes, are the hallmarks of diabetic cheiroarthropathy (DCA) [12]. We found that shoulder capsulitis and cheiroarthropathy were two outcomes with a high frequency rate of 9.3% in our analysis.

Muluneh et al. [13] highlighted the worldwide importance of diabetes by discovering a high prevalence of adhesive capsulitis and decreased joint mobility among diabetic individuals in Ethiopia.

Diabetes makes a number of rheumatologic symptoms worse, such as trigger finger, rotator cuff tears, frozen shoulder, Dupuytren's contracture, cheiroarthropathy in the upper limb, and Achilles tendinopathy and plantar fasciitis in the lower limb [14]. These conditions can limit the range of motion in the afflicted joint, which can impair function and everyday activities. There have been reports of severe Achilles tendon damage, including lower leg plantar fasciitis, which limits ankle range of motion and causes diabetic foot ulcers [15]. The results of this investigation showed that decreased joint mobility was seen in 8% of the DM patients examined.

Dupuytren's contracture has been linked to an increased risk of other disorders, including DM [16]. The prevalence of Dupuytren's contracture was reported to be 6.7% among the examined patients.

The prevalence of trigger finger was 4.1% in this study. The prevalence of trigger finger in DM patients is 3.2%, per Löfgren et al. [17]. Approximately 1%-2% of the population is impacted by TF. Diabetes-related prevalence rates and risk for TF range from 1.5% to 20%, depending on the demographic [18,19].

Charcot neuroarthropathy (CN) is an uncommon, degenerating condition that mainly affects people with diabetes. It is sometimes referred to as neuropathic osteoarthropathy, Charcot foot, or just Charcot joint [20,21]. Charcot foot affected 3% of the individuals in the study. Myocardial illness in diabetic people that is not brought on by CAD, hypertension, or other heart disorders is known as diabetic cardiomyopathy [22,23]. It was found that 5.8% of the individuals assessed in this study had diabetic myopathy.

According to this study, obesity, advanced age, and the length of diabetes are all important predictors of MSDs. These results are consistent with recent systematic reviews and meta-analyses, including one by Azami et al., which showed that individuals with greater BMI and longer duration of diabetes are more likely to develop MSDs [24].

The study’s strengths included a sizable sample size, focusing specifically on type 2 DM patients, and concentrating on the MSK system, one of the most important systems.

Strengths of the study

The study has several strengths. It included a large sample size, and the methodology clearly articulated the study’s purpose. The study focused specifically on patients with type 2 diabetes and targeted the MSK system, which is an important and underexplored area. To the best of our knowledge, few studies have investigated this topic in the Western Region of Saudi Arabia, highlighting the novelty and relevance of our work.

Limitations

A limitation of the present study was the use of a self-administered questionnaire, which may have introduced recall bias. Another limitation was the cross-sectional study design, which allows for identifying associations between variables but does not establish causal relationships. The predominance of females in the study could also be a source of bias, as females are more prone to MSK disorders than males. Additionally, a potential selection bias may have arisen from the underrepresentation of individuals without social media apps, such as elderly patients, which could have skewed the average age. Furthermore, reliance on self-reported diagnoses without clinical verification and incomplete acknowledgment of selection bias were limitations that should be noted to improve transparency and interpretability.

## Conclusions

OA was the most common MSK consequence among the diabetic patients in this study, with a prevalence of 31.9%. Patients who had been married for a longer period of time, had a higher mean BMI, had had diabetes mellitus for 1-10 years, and had any macrovascular complications - aside from stroke - were much more likely to have MSK abnormalities. Multivariate logistic regression analysis found that having any of the DM complications or having a diabetic foot was a risk of MSK disorders among the studied patients. For prompt and effective treatment, it is essential to identify MSK disease symptoms in diabetic patients as soon as possible. To avoid incapacity and a detrimental effect on patients' quality of life, the supervising family doctors should be informed about the possible repercussions of MSK and the importance of early detection.

## Appendices

**Table 7:** Sociodemographic data

Sociodemographic Data	Answers
Age (in years)	
Gender	Male/Female
Region	Western Region/Eastern Region/Central Region/Southern Region
Educational level	High school or below/Diploma or equivalent/Bachelor's degree/Master's degree/PhD degree
Employment	Employed/Unemployed/Student/Retired
Marital status	Single/Married/Divorced/Widowed

**Table 8:** Medical history

Medical History	Answers
Type of DM	T1DM/T2DM
Duration of DM (in years)	
HbA1c	
Height (in cm)	
Weight (in kg)	
Have you received any education or information about the relationship between diabetes and musculoskeletal health?	Yes/No
General Complications Among DM Patients	
Please indicate any of the following macrovascular complications you have previously encountered. Please select all that apply:	Coronary Artery Disease (CAD)/Peripheral Arterial Disease (PAD)/Aortic Aneurysm/Stroke
Please specify if you have experienced any of the following complications related to diabetes. Select all that apply:	Retinopathy/Nephropathy/Neuropathy/Dyslipidemia/Diabetic foot
MSK Complications Among DM Patients	
Have you ever been diagnosed with osteoarthritis?	Yes/No
Have you ever been diagnosed with shoulder capsulitis? (This condition, also known as adhesive capsulitis or frozen shoulder, is characterized by stiffness, pain, and limited range of motion in the shoulder joint.)	Yes/No
Have you ever been diagnosed with Carpal Tunnel Syndrome? A disorder that affects the hand and wrist, resulting from compression of the median nerve, and can cause symptoms such as tingling, numbness, and weakness in the affected hand.	Yes/No
Have you ever been diagnosed with Dupuytren's contracture? This condition causes one or more fingers to bend into the palm, making it difficult to straighten them.	Yes/No
Have you ever been diagnosed with Cheiroarthropathy? Thickening and tightening of the skin on the hands, leading to reduced mobility and flexibility.	Yes/No
Have you ever been diagnosed with trigger finger? Trigger finger, also known as stenosing tenosynovitis, is a condition where one of your fingers gets stuck in a bent position and may straighten with a snap, like pulling and releasing the trigger of a gun.	Yes/No
Have you ever been diagnosed with limited joint mobility? Limited joint mobility is a condition characterized by restricted movement in one or more joints, commonly observed in individuals with diabetes mellitus. affects the hands and fingers, accompanied by stiffness, and reduced flexibility.	Yes/No
Have you ever been diagnosed with Charcot joint? This is a deformity and functional impairment, often occurring in the foot or ankle due to nerve damage and loss of sensation in a joint.	Yes/No
Have you ever been diagnosed with diabetic myopathy? There is weakness, atrophy, and changes in muscle structure and function.	Yes/No
Have you ever been diagnosed with diabetic osteoporosis or any diabetic-related bone issues?	Yes/No
